# HLA Class I Downregulation in Progressing Metastases of Melanoma Patients Treated With Ipilimumab

**DOI:** 10.3389/pore.2022.1610297

**Published:** 2022-04-22

**Authors:** Andrea Ladányi, Barbara Hegyi, Tímea Balatoni, Gabriella Liszkay, Raphael Rohregger, Christoph Waldnig, József Dudás, Soldano Ferrone

**Affiliations:** ^1^ Department of Surgical and Molecular Pathology, National Institute of Oncology, Budapest, Hungary; ^2^ Department of Thoracic and Abdominal Tumors and Clinical Pharmacology, National Institute of Oncology, Budapest, Hungary; ^3^ Doctoral School of Pathological Sciences, Semmelweis University, Budapest, Hungary; ^4^ Department of Oncodermatology, National Institute of Oncology, Budapest, Hungary; ^5^ Department of Otorhinolaryngology and Head and Neck Surgery, Medical University of Innsbruck, Innsbruck, Austria; ^6^ Department of Surgery, Massachusetts General Hospital and Harvard Medical School, Boston, MA, United States

**Keywords:** immunotherapy, melanoma, ipilimumab, HLA class I expression, longitudinal study

## Abstract

Characterization of the molecular mechanisms underlying antitumor immune responses and immune escape mechanisms has resulted in the development of more effective immunotherapeutic strategies, including immune checkpoint inhibitor (ICI) therapy. ICIs can induce durable responses in patients with advanced cancer in a wide range of cancer types, however, the majority of the patients fail to respond to this therapy or develop resistance in the course of the treatment. Information about the molecular mechanisms underlying primary and acquired resistance is limited. Although HLA class I molecules are crucial in the recognition of tumor antigens by cytotoxic T lymphocytes, only a few studies have investigated the role of their expression level on malignant cells in ICI resistance. To address this topic, utilizing immunohistochemical staining with monoclonal antibodies (mAbs) we analyzed HLA class I expression level in pre-treatment and post-treatment tumor samples from melanoma patients treated with ipilimumab. Twenty-nine metastases removed from six patients were available for the study, including 18 pre-treatment and 11 post-treatment lesions. Compared to metastases excised before ipilimumab therapy, post-treatment lesions displayed a significantly lower HLA class I expression level on melanoma cells; HLA class I downregulation was most marked in progressing metastases from nonresponding patients. We also evaluated the level of infiltration by CD8^+^ T cells and NK cells but did not find consistent changes between pre- and post-treatment samples. Our results indicate the potential role of HLA class I downregulation as a mechanism of ICI resistance.

## Introduction

Immune checkpoint inhibitor (ICI)-based therapy has brought major breakthrough in cancer treatment, becoming the mainstream of treatment for many cancer types. The first such agent was the anti-CTLA-4 (cytotoxic T lymphocyte-associated antigen 4) monoclonal antibody ipilimumab, which was approved for treatment of patients with advanced melanoma in 2011 [1, 2], later followed by antibodies blocking PD-1 (programmed death receptor 1) or its ligand, PD-L1 (programmed death ligand 1). These monoclonal antibodies have induced impressive clinical responses in a small proportion of patients in a broad spectrum of cancer types. However, the majority of patients do not respond or develop resistance to these immunotherapeutic agents. The mechanisms of primary and acquired resistance are poorly understood. Many potential biomarkers have been proposed that could predict the efficacy of ICI therapies. They include, among others, PD-L1 expression by tumors when PD-1- or PD-L1-specific mAbs are used, tumor mutational burden (TMB), neoantigen load, microsatellite instability, tumor infiltration by immune cells and immune-related gene expression in tumors [3, 4]. Moreover, many potential mechanisms underlying acquired resistance to ICI-based therapy have been identified [3–5]. They include neoantigen loss [6], loss of PTEN expression and activation of β-catenin [7], mutations in JAK1/2 leading to defects in the IFN signaling pathway, mutations in beta-2 microglobulin (B2M), the light chain of HLA class I antigens, resulting in defective HLA class I antigen presentation [8–10], or upregulation of other immune checkpoints such as TIM-3, LAG-3 or VISTA [7, 9]. The same mechanisms have also been implicated in primary resistance to ICI-based therapy [3, 4, 8, 10, 11].

The efficacy of ICI-based therapy depends on the recognition of tumor antigens by cognate cytotoxic T lymphocytes in the context of human leukocyte antigen (HLA) class I molecules. The key role played by HLA class I molecules may account for the described associations of some of their characteristics with response to checkpoint blockade-based therapy. They include the association of maximal heterozygosity of HLA-I loci as well as high evolutionary divergence of HLA class I genotype with improved survival following ICI-based therapy [12, 13], and the association of high degree of HLA-I promiscuity with reduced survival and lower response rate in patients receiving ICIs [14], in addition to the mentioned primary or acquired resistance to anti-PD-1 mAb-based therapy in patients with structural mutations or loss of heterozygosity (LOH) of B2M [8–10]. The frequency of defects in HLA class I antigen processing machinery (APM) component expression and/or function caused by structural mutations is low [15, 16] and therefore has limited clinical relevance. In contrast, the frequency of defects in HLA class I APM component expression and/or function caused by epigenetic mechanisms and/or transcription dysregulation is high in most, if not all cancer types analyzed [15–17]. Nevertheless only a few studies have assessed the value of HLA class I expression level as a biomarker to predict the clinical responses to ICI-based therapy and have found an association between these two parameters [18, 19]. Furthermore, low gene or protein expression of the HLA class I APM components has been described in progressing lesions in some patients with melanoma, lung cancer, or Merkel cell carcinoma treated with ICI-based therapy [7, 9, 10, 20]. These results have been mainly obtained in patients treated with anti-PD-1 mAb; to the best of our knowledge, no information is available about melanoma patients treated with ipilimumab. In a recent study we have shown that tumor cell HLA class I expression level in pre-treatment samples of melanoma patients is a biomarker of clinical response to ipilimumab therapy and of patients’ survival [19]. To explore potential changes in HLA-I expression level in ipilimumab-treated patients, in the present investigation we have assessed HLA class I expression level on melanoma cells in pre- and post-treatment metastases removed from patients treated with ipilimumab. Since effective tumor antigen recognition relies on the interaction between CD8^+^ cytotoxic T lymphocytes and HLA class I molecules while HLA-I negative tumors may be sensitive to killing by natural killer (NK) cells, we also examined infiltration of pre- and post-treatment tumor samples by CD8^+^ T cells and NK cells.

## Materials and Methods

### Tumor Samples

We obtained archived paraffin blocks of sequential (pre- and post-treatment) tissue samples of patients with metastatic melanoma treated with ipilimumab between 2010 and 2015. Sample collection was restricted to metastases surgically removed within a 2 years range before or after ipilimumab treatment; 29 metastases of six patients were available for the study. The clinical characteristics of the patients are shown in Table 1. TNM classifications and stage grouping criteria were based on the 7th Edition of AJCC Staging System. Five of the six patients received systemic treatment before ipilimumab therapy; all of them had chemotherapy while two also received radiotherapy, and one patient (Pt3) had already received ipilimumab therapy 32 months before the ipilimumab reinduction treatment evaluated in the present study. Responses to therapy were evaluated based on immune-related response criteria (irRC) [21]. One patient (Pt1) was scored as complete response (CR) with a few residual cutaneous papules, which showed minimal progression 11 months following initiation of ipilimumab therapy and were excised. Pt2 achieved stable disease (SD) lasting for 10 months, while Pt 3 showed short-term SD lasting for 4 months; the other three patients exhibited progressive disease (PD). Pt1 and Pt2 were classified as responders while the other four patients as nonresponders in the analysis. Progression-free survival (PFS) and overall survival (OS) were calculated from the commencement of ipilimumab treatment till the last follow-up, tumor progression or death, respectively. Altogether 29 metastases were studied, 18 pre-treatment and 11 post-treatment surgical samples (Table 1). Of the post-treatment samples, three were residual metastases from Pt1 while the other eight were progressing lesions.

**TABLE 1:** Patient and sample characteristics.

	Age (years)	Gender	Stage	ECOG Status	BRAF Status	BOR	PFS (months)	OS (months)	Pre samples analyzed	Post samples analyzed
Pt1	52	Female	III N3c	0	mut	CR	11	67+	2 (cut.)	3 (cut./sc.—residual)
Pt2	51	Female	IV M1c	0	mut	SD	10	43	1 (sc.)	1 (sc.—progression)
Pt3	73	Male	IV M1a	0	wt	SD	4	42	4 (LN, cut./sc.)	2 (LN, sc.—progression)
Pt4	53	Female	IV M1b	0	mut	PD	4	29	3 (LN, sc.)	3 (sc.—progression)
Pt5	59	Male	IV M1c	1	wt	PD	3	9	1 (sc.)	1 (cut.—progression)
Pt6	57	Female	IV M1c	0	mut	PD	3	8	7 (LN, breast)	1 (LN—progression)

ECOG, Eastern Cooperative Oncology Group; mut, mutant; wt, wild type; BOR, best overall response; CR, complete response; SD, stable disease; PD, progressive disease; PFS, progression-free survival; OS, overall survival; Pre, pre-treatment; Post, post-treatment; cut., cutaneous; sc., subcutaneous; LN, lymph node.

### Monoclonal Antibodies

The mouse monoclonal antibody (mAb) HCA2, recognizing B2M-free HLA-A (excluding -A24), -B7301, and -G heavy chains, the mAb HC10, which recognizes B2M-free HLA-A3, -A10, -A28, -A29, -A30, -A31, -A32, -A33, HLA-B (excluding -B5702, -B5804, and -B73), and HLA-C heavy chains and the B2M-specific NAMB-1 were developed and characterized as described [22, 23]. The mouse anti-human CD8 mAb and the mouse anti-human NKp46 mAb were purchased from Dako (Glostrup, Denmark) and from R&D Systems (Abingdon, United Kingdom), respectively.

### Immunohistochemical Staining of Tumor Tissue Sections

Immunohistochemical staining of tissue sections of formalin-fixed, paraffin-embedded tumor samples was performed as described earlier [19, 24]. Briefly, deparaffinated sections were treated with 3% H_2_O_2_ in methanol to block endogenous peroxidases, then antigen retrieval was performed by heating at 98°C for 40 min in citrate buffer (pH 6.0), followed by incubation with protein blocking solution (Protein Block, Serum-Free, Dako) for 10 min at room temperature, and incubation with the primary antibodies overnight at 4°C. For staining detection the SS™ One-Step Polymer-HRP IHC Detection System (BioGenex, Fremont, CA) and 3-amino-9-ethylcarbazole (AEC; Vector Laboratories, Inc., Burlingame, CA) were used followed by counterstaining with hematoxylin. In the case of labeling with anti-HLA class I mAbs, the percentage of the area displaying stained melanoma cells was determined in the metastases. Intratumoral density of CD8^+^ and NKp46^+^ lymphocytes was assessed as described earlier [24]; briefly, the number of labeled cells was counted within the metastases in at least 10 (median: 35, range: 10–120) randomly chosen fields per sample, using a graticule of 10 × 10 squares designating an area of 0.0625 mm^2^ at ×400 magnification. For patients with more than one metastasis available the average values were also calculated for each marker, separately for pre- and post-treatment samples. The statistical significance of the differences between pre- and post-treatment samples was determined using the Mann-Whitney U test.

### Computerized Analysis of the Staining Intensity by Anti-HLA Class I Antibodies

The immunohistochemistry slides were acquired in TissueFaxs brightfield (Tissuegnostics, Vienna, Austria) system with a ×40 magnification dry lens coupled onto a Zeiss Axio Imager Z2 Microscope (Jena, Germany) and an eight slide automatic stage (Märzenhäuser, Wetzlar, Germany) using a Pixelink camera (Pixelink, Rochester, NY, United States). Regions of interest containing metastases without obvious artifacts were selected (Supplementary Figure S1) and analyzed using the HistoQuest (TissueGnostics) image cytometry software. “Cells” were identified on the basis of the hematoxylin stained nuclei and the immunohistochemical reaction was identified by ring mask (Supplementary Figure S2) [25]. The cell nuclei area was used to distinguish among cell populations (Supplementary Figure S3). The staining signal was quantified using a single-reference-shade color deconvolution algorithm [26]. Quantifications were confirmed visually by the backward connection function of the HistoQuest program (Supplementary Figures S3, S4).

## Results

**FIGURE 1:**
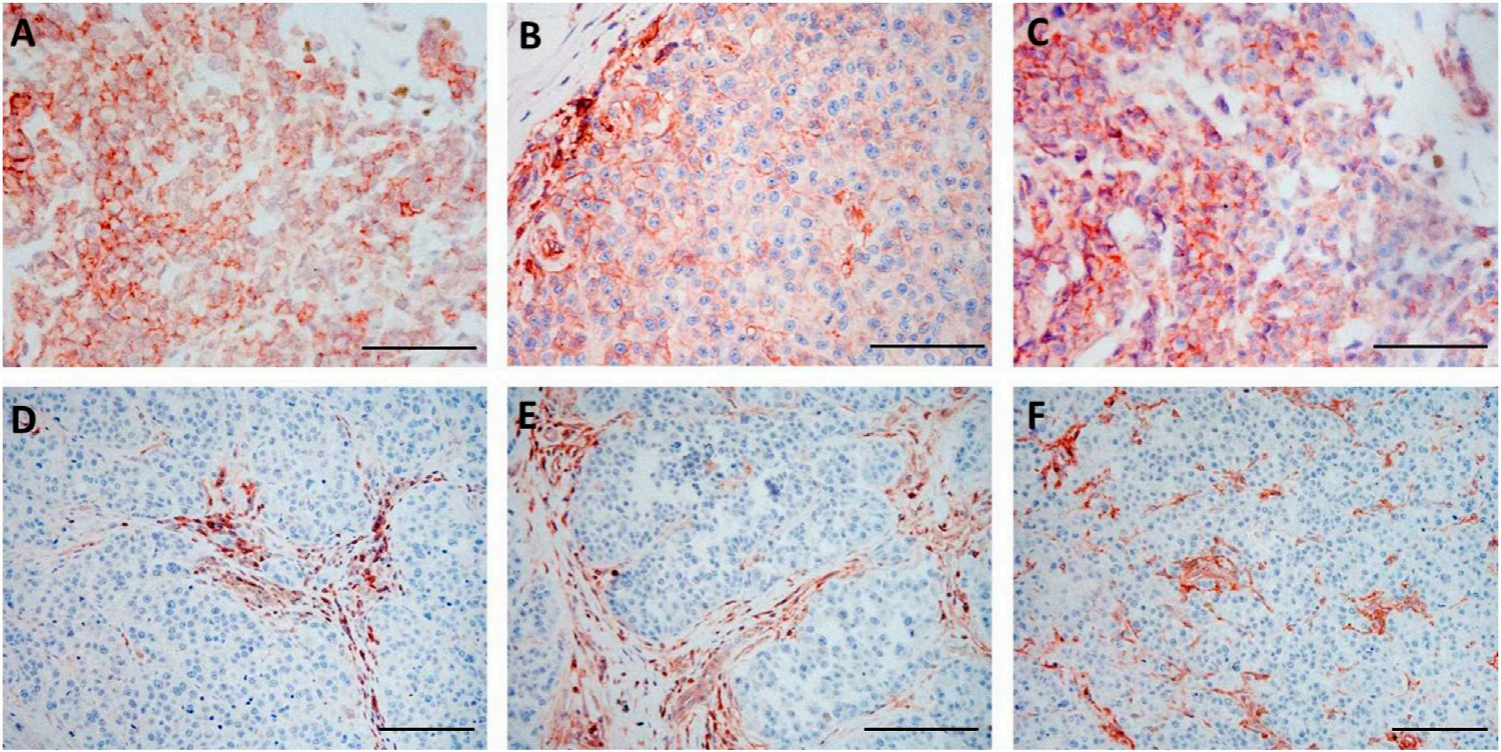
Immunohistochemical staining of pre-treatment **(A–C)** and post-treatment **(D–F)** samples from the same patient (Pt3) with HLA-A heavy chain-specific mAb HCA2 **(A,D)**, HLA-B,C heavy chain-specific mAb HC10 **(B,E)** and B2M-specific mAb NAMB-1 **(C,F)** (3-amino-ethylcarbazole, red). Scale bars: 100 μm.

**FIGURE 2:**
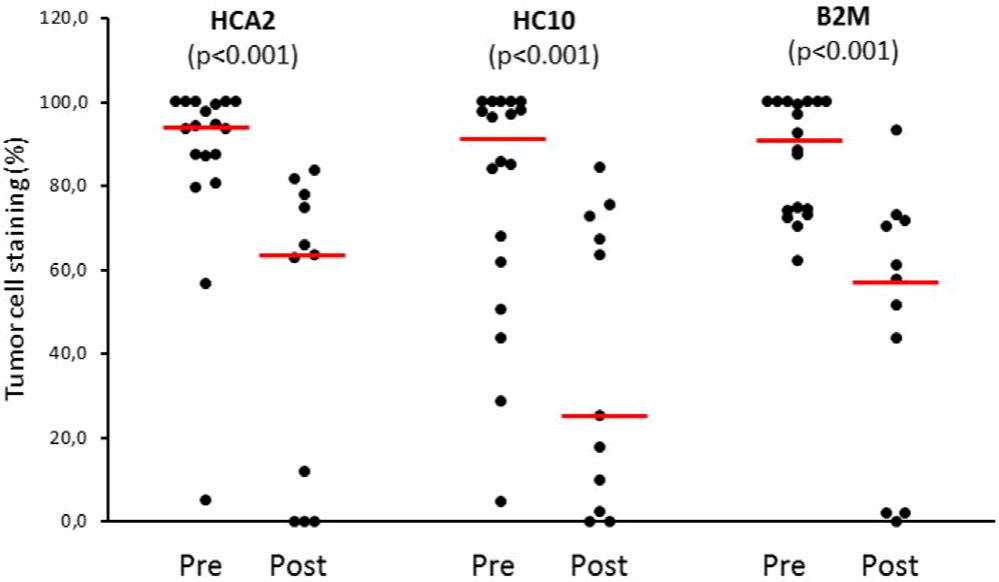
HLA class I expression of melanoma cells (% of stained area) in pre-treatment (Pre, *n* = 18) and post-treatment (Post, *n* = 11) metastases from ipilimumab-treated patients. Circles: percentage values of individual samples; horizontal line: median.

Utilizing IHC staining with mAbs we analyzed the expression of HLA class I subunits in sequential metastasis samples from six melanoma patients treated with ipilimumab; the samples analyzed included 18 pre- and 11 post-treatment surgically excised metastases (Table 1). Comparing pre-treatment and post-treatment samples of all patients evaluated together, the expression of HLA class I subunits, as measured by the % of stained melanoma cells, was significantly lower in post-treatment metastases compared to pre-treatment ones (Figures 1, 2). The medians and ranges of the percentage values of melanoma cells stained by HLA-A heavy chain-specific mAb HCA-2, by HLA-B,C heavy chain-specific mAb HC10 and by anti-B2M mAb NAMB-1 were 94.0 (5.1–100), 91.0 (4.5–100) and 90.5 (62.2–100) in the pre-treatment metastases, and 63.5 (0–83.6), 25.0 (0–84.2) and 57.6 (0–93.1) in the post-treatment metastases, respectively. The percentage of melanoma cells stained by all three mAbs tested was higher than 80 in the majority of the 18 pre-treatment metastases analyzed, compared to only 1 of the 11 post-treatment metastases. In agreement with our previous results [19], metastases with a heterogenous staining pattern displayed higher labeling at the margin of the tumors in the proximity of inflammatory cells, consistent with locally induced expression. Percentages of staining with the three antibodies were fairly consistent in the majority of cases, with discrepancies larger than 30% in only 7 of the 29 metastases. Furthermore, comparing tumor cell staining of different lesions with the same antibody, we detected a moderate level of intrapatient heterogeneity in most patients in the case of pre-treatment metastases and in two of the three patients with more than one post-treatment lesions (Supplementary Table S1).

**FIGURE 3:**
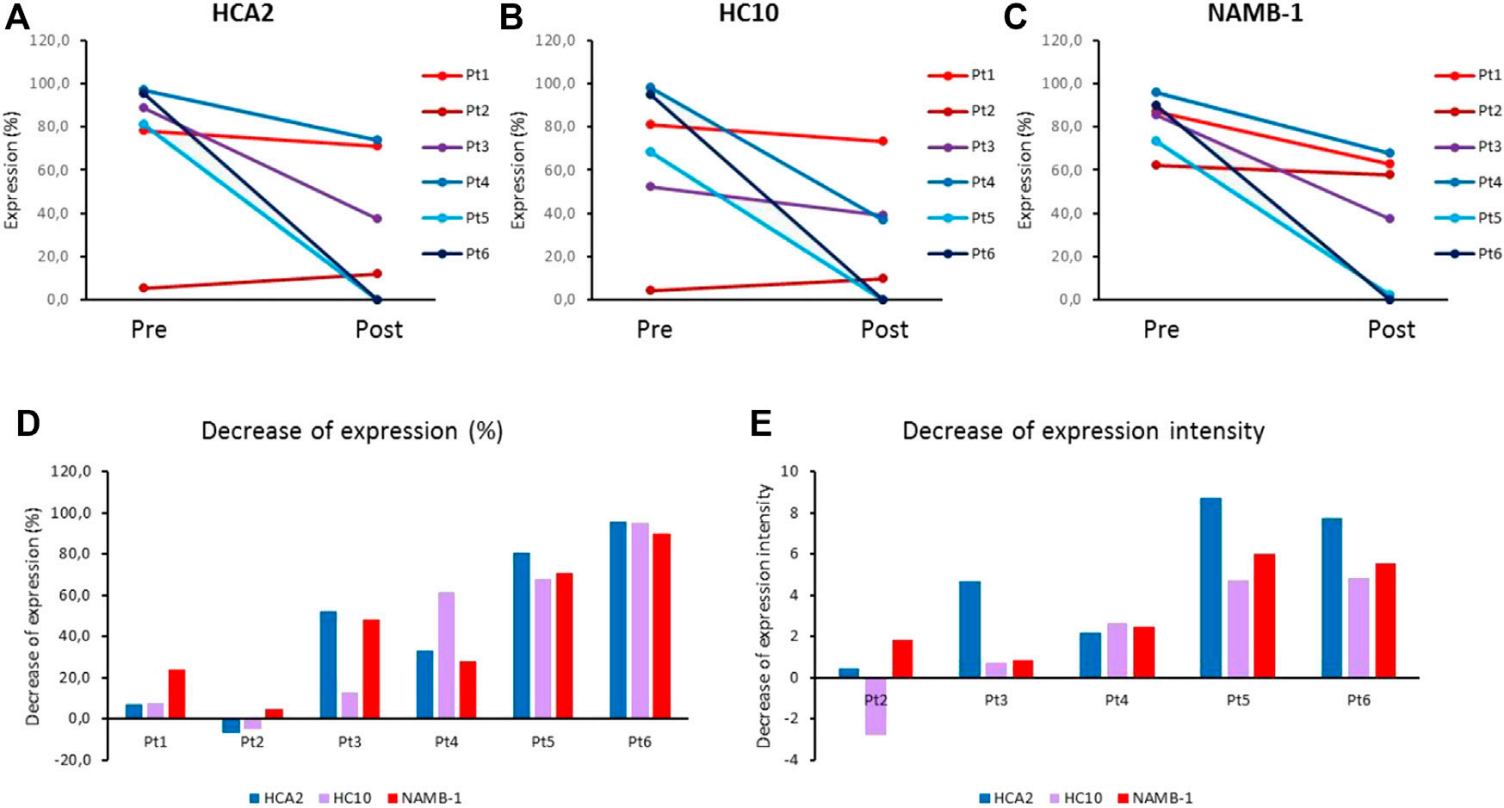
**(A–C)** Average HLA class I expression of melanoma cells (% of stained area) in pre-treatment (Pre) and post-treatment (Post) metastases from ipilimumab-treated patients, labeled by HLA class I subunit-specific mAbs HCA2 **(A)**, HC10 **(B)** and NAMB-1 **(C)**. **(D,E)** Decrease of mean expression (% of stained area) **(D)** and of staining intensity **(E)** in post-treatment metastases as compared to autologous pre-treatment metastases from ipilimumab-treated patients, stained by HLA class I subunit-specific mAbs.

Comparison of the HLA class I subunit expression levels in pre- and post-treatment metastases removed from each individual patient revealed HLA-I downregulation mainly in the case of progressing lesions of nonresponding patients; in contrast, minimal or no change was found in responding patients (Pt1 and Pt2). Interestingly, in Pt1 exhibiting the best overall response and long-term survival, the baseline HLA class I subunit expression was high in the pre-treatment metastases and remained high in the post-treatment (residual) metastases (Figures 3A–C, Supplementary Table S1). In contrast, HLA class I subunit downregulation was maximal in the metastases from Pt5 and Pt6 exhibiting the shortest PFS and OS (Figure 3D, Supplementary Table S1). Results of quantitative evaluation of staining intensity in representative pre- and post-treatment samples of nonresponding patients with progressing lesions are in agreement with this finding (Figure 3E).

Since the efficacy of immune checkpoint inhibitors depends on the recognition of tumor antigen derived peptides by cytotoxic T lymphocytes in the context of HLA class I proteins, we also examined the extent of infiltration of CD8^+^ T cells in pre-treatment vs. post-treatment tumors. The infiltration showed considerable intertumor variability and did not exhibit any consistent change between pre- and post-treatment time points (Supplementary Figure S5). Furthermore, the extent of NK cell infiltration was also tested because these cells are known to recognize HLA class I negative cells so their activity could possibly complement that of CD8^+^ T lymphocytes. Using NKp46 as a NK cell marker, we detected a very low number of NK cells infiltrating both pre-treatment and post-treatment tumors; furthermore, no significant difference could be found between the two sample sets (Supplementary Figure S5).

## Discussion

Many currently used antitumor immunotherapeutic modalities rely on T cell recognition of tumor antigens, in which presentation by HLA class I antigens has a crucial role. It is also an important factor in spontaneous (not therapy-induced) immunity against tumors, which is reflected by the reported association of defects of HLA class I APM component expression with immune escape, disease progression and poor prognosis in several tumor types [16, 17, 27]. Moreover, B2M aberrations have been implicated as resistance mechanisms in patients treated with different T-cell based immunotherapies [28–30] or with immune checkpoint inhibitors [8–10]. However, genomic loss of B2M occurs infrequently, and there is little information about the role of other possible causes of decreased B2M and HLA class I expression in unresponsiveness or acquired resistance to ICIs.

Results of studies on associations of gene alterations or loss of HLA class I APM components with response to ICIs are equivocal. While mutations or LOH of B2M were described in some patients exhibiting primary or acquired resistance to PD-1 inhibitors [8–10], other recent studies did not find an association between LOH in B2M or HLA class I loci and response to anti-PD-1/PD-L1 agents [31, 32]. Moreover, pre-treatment HLA class I gene expression or mutational status did not differ in responders vs. nonresponders to ipilimumab [33]. On the other hand, an APM score composed of eight APM-associated genes (including B2M) predicted response to anti-PD-1/PD-L1 agents [34]. Furthermore, HLA genes were found to be upregulated in on-therapy samples of responders, but downregulated in nonresponders in melanoma patients treated with anti-PD-1 therapy [35].

Few studies have examined protein expression of HLA class I molecules in patients receiving ICI therapy. A study on metastatic melanoma patients treated with ICI therapies [10] found decreased B2M and/or HLA class I expression in some patients harboring B2M gene alterations and showing primary or acquired ICI resistance. Another report on metastatic melanoma [7] described downregulation of HLA-A expression in biopsies of progressing lesions compared to pre-treatment ones in 4 of 18 melanoma patients receiving ICI therapy [7]. However, no significant alterations in gene or protein expression of HLA class I were found in progressing tumors in six patients with different types of carcinomas [36]. A recent study [37] demonstrated low HLA class I expression in 40% of pre-treatment and 31% of progression melanoma tumors, and no association with response to PD-1 inhibition. Similarly, no association between HLA class I expression and response to anti-PD-1 therapy was found in melanoma patients in another study, although it proved predictive of response to anti-CTLA-4 treatment [18]. The results of our previous study corroborated the role of HLA class I expression in influencing the efficacy of ipilimumab [19].

In the present work, we analyzed HLA class I tumor cell expression as well as CD8^+^ T cell and NK cell infiltration in longitudinal tumor samples from a subset of patients with available pre-treatment and post-treatment surgically removed metastases. Analyses of longitudinal tumor samples from different stages of treatment are necessary for better understanding of the mechanisms of response or resistance to this type of therapy [3]. Most such studies performed so far have focused mainly on characterization of early on-treatment tumor biopsies, yielding important information regarding the biological effects of ICI therapies as well as predictive biomarkers [35, 38–42] while few studies aimed at investigating tumors progressing after or on ICI therapy, especially in case of CTLA-4 inhibitors [43]. To the best of our knowledge, ours is the first study interrogating HLA class I expression longitudinally in tumor samples of patients treated with ipilimumab. We found decreased tumor cell expression in the majority of progressing metastases of all nonresponding patients; this decrease was most marked in the case of patients with the worst prognosis, although a statistical analysis of the correlation with survival could not be performed because of the limited number of patients tested. Nevertheless, these results support the hypothesis of immunoediting in patients receiving ipilimumab treatment, resulting in HLA class I loss and in tumor progression. This process has been described in the case of acquired resistance to immunotherapy, but a low level of antitumor immune activity may be present even in clinically nonresponding patients, which could shape the immunogenicity of the progressing tumors. Unfortunately, we had only one responding patient with residual (minimally progressing) metastases: therefore solid conclusions could not be drawn from our study. Nonetheless, it is worth mentioning that HLA class I expression in both pre-treatment and post-treatment tumors was consistently high in this patient, implicating lack of immunoediting, at least regarding HLA class I expression.

We also examined potential changes in infiltration level of two types of immune effector cells, CD8^+^ T lymphocytes and NK cells, both of which were found associated with clinical response to ipilimumab in our previous study on pre-treatment metastatic samples [24]. The density of CD8^+^ T cells showed considerable variability among metastases, even in the same patient, and no consistent difference could be observed between pre-treatment and post-treatment samples. Similarly, no significant pre-treatment/post-treatment change could be found in the case of NK cells; the latter were not detectable or present in only a low number in most of the metastases examined, in agreement with the information in the literature [24, 44, 45]. Investigations on longitudinal samples from melanoma patients receiving anti-PD-1 mAbs found elevated infiltration level of CD8^+^ T cells in on-treatment samples of responding patients [35, 40, 41, 46], while few and uncertain data are available on progressing cases [41]. In a recent study higher density of NK cells was observed in pre-treatment and early during treatment tumor samples of responders compared to nonresponders [45]. Few reports have been published on longitudinal studies in melanoma patients treated with ipilimumab. A study on advanced melanoma patients demonstrated increase in tumor-infiltrating lymphocytes in early on-treatment biopsies of patients benefiting from the therapy, but no significant change in the number of CD8^+^ T cells [38].

We recognize the inherent limitations of our study caused by its retrospective nature and also by the limited number of cases with available pre-treatment and post-treatment surgical samples. However, there are few reports of longitudinal studies on local immunological features of patients receiving immune checkpoint inhibitor therapy, especially in the case of ipilimumab, and most of them encompass a relatively small sample size. The findings of our pilot study will require validation in future prospective studies involving larger patient cohorts enabling more complex statistical analysis. A strength of our analysis, on the other hand, is that it was performed on whole sections from surgical samples; therefore the results we have presented are expected to be more reliable than those generated by the analysis of biopsies, given the known heterogeneous distribution within tumors of both HLA antigens and immune cells [47, 48].

In conclusion, we found a decreased HLA class I expression level by malignant cells in post-treatment progressing metastases of melanoma patients receiving ipilimumab therapy compared to pre-treatment metastatic samples. This finding was a consistent feature in our cohort of patients with progressing tumors, but was not observed in the residual metastases of a responding patient. Further work is warranted to validate these findings in larger patient cohorts, as well as to explore whether HLA class I loss represents a common mechanism of primary and acquired resistance to immune checkpoint inhibitors as well as other T-cell based immunotherapeutic modalities in melanoma and other cancer types. Accumulating evidence on immunologic changes observed in longitudinal studies of patients receiving immunotherapy will contribute to an improved understanding of the molecular mechanisms underlying resistance to such therapies and may help to find appropriate strategies to overcome them [3–5].
