# Supplementary material for: HLA Class I Downregulation in Progressing Metastases of Melanoma Patients Treated With Ipilimumab
Source: Pathol Oncol Res. 2022 Apr 22;28:1610297. doi: 10.3389/pore.2022.1610297 (PMC9073691; doi:10.3389/pore.2022.1610297)
Supplement: Supplementary file 1 [file DataSheet2.PDF]

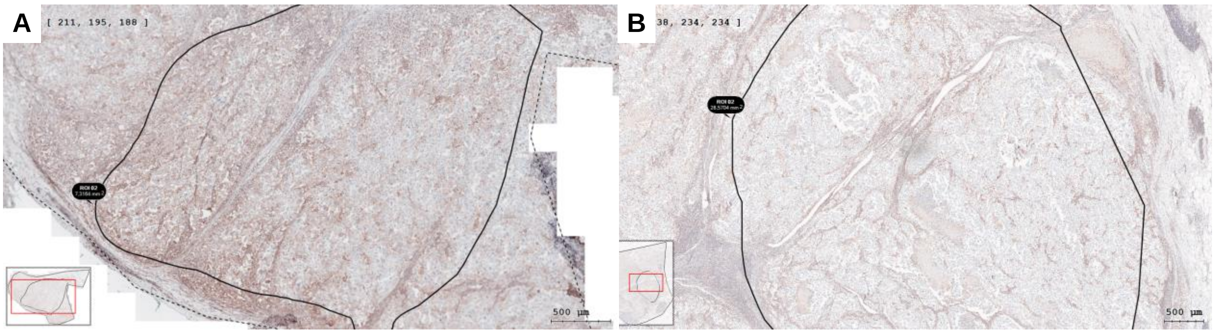

Supplementary Figure 1. Overview of HCA2 staining in pre-therapy (A) and in post-therapy sample (B)

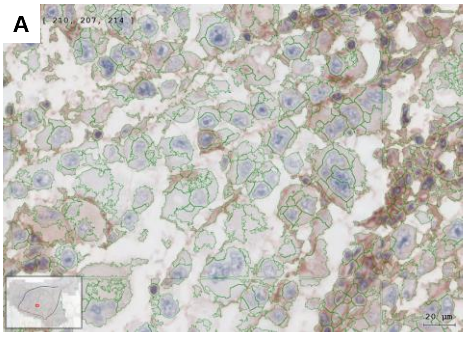

B

Nuclear Segmentation	
Nuclei Size	130
Remove small-sized objects	3
Remove weakly stained objects	2
Automatic Background Threshold	No
Background Threshold	8
Virtual Channel	no
Post Processing Order	Remove, Merge
Remove Labels	No
Use Merging Rules	No

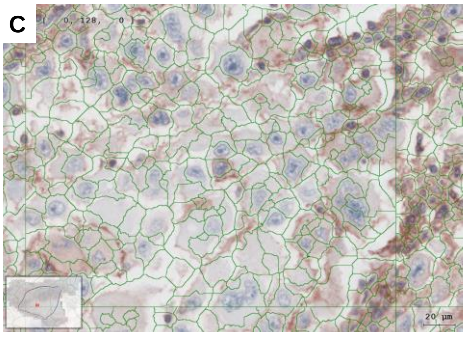

D

Cellular Segmentation	
Use Ring Mask	Yes
Interior Radius	-0,14 µm
Exterior Radius	9 µm
Use Identified Cell Mask	Outside
Max Growing Steps	0,55 µm
Skip Steps	0 µm
Use Nuclei Mask	Yes
Automatic Background Th...	No
Background Threshold	8

Supplementary Figure 2. A) Recognition of the cell nuclei based on their hematoxylin staining. B) Cell nuclear segmentation parameters. C) Recognition of the HCA2 staining by ring mask cellular mask detection. D) Cellular mask segmentation parameters

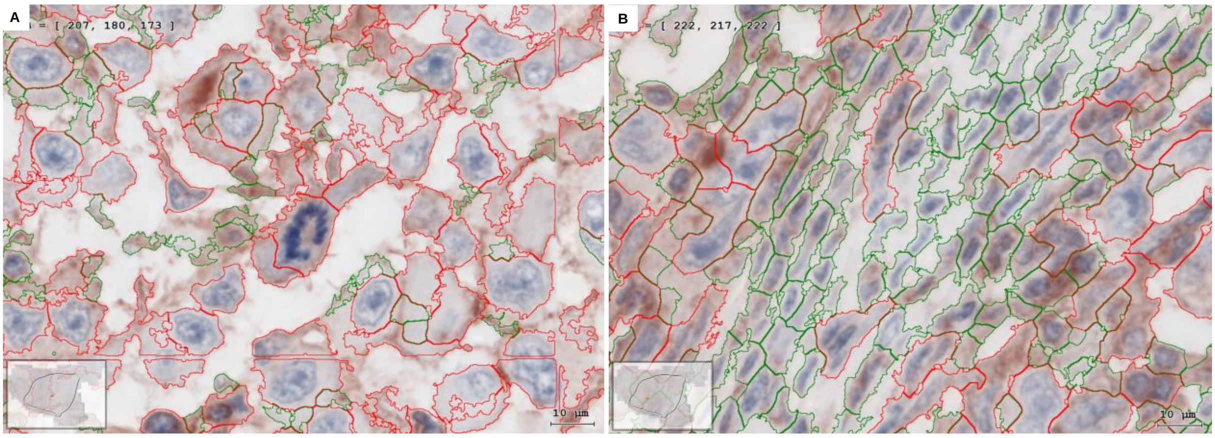

Supplementary Figure 3 A and B. Use of cell nuclei area to distinguish small and large cells. Backward connection represents large cells in red, and small cells in green

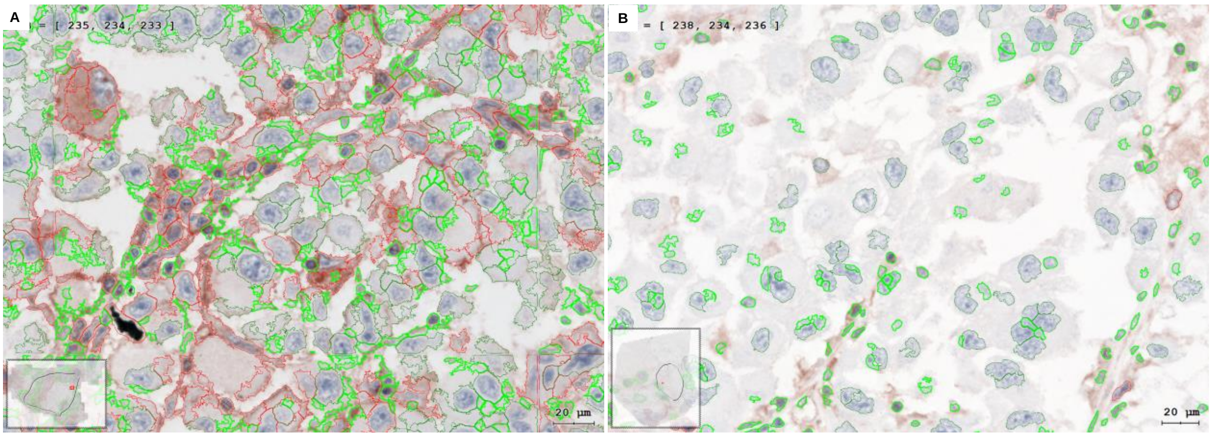

Supplementary Figure 4. Comparison of HCA2 staining intensity before (A) and after therapy (B). Large cells with obvious HCA2 staining are marked with red. Large cells without obvious HCA2 staining are marked with dark green. Light green marker shows small cells that were excluded by cell nuclear area distinguishing and were not considered. The intensity recognition confirms the obvious staining intensity difference
