# Supplementary material for: HLA Class I Downregulation in Progressing Metastases of Melanoma Patients Treated With Ipilimumab
Source: Pathol Oncol Res. 2022 Apr 22;28:1610297. doi: 10.3389/pore.2022.1610297 (PMC9073691; doi:10.3389/pore.2022.1610297)
Supplement: Supplementary file 2 [file DataSheet3.PDF]

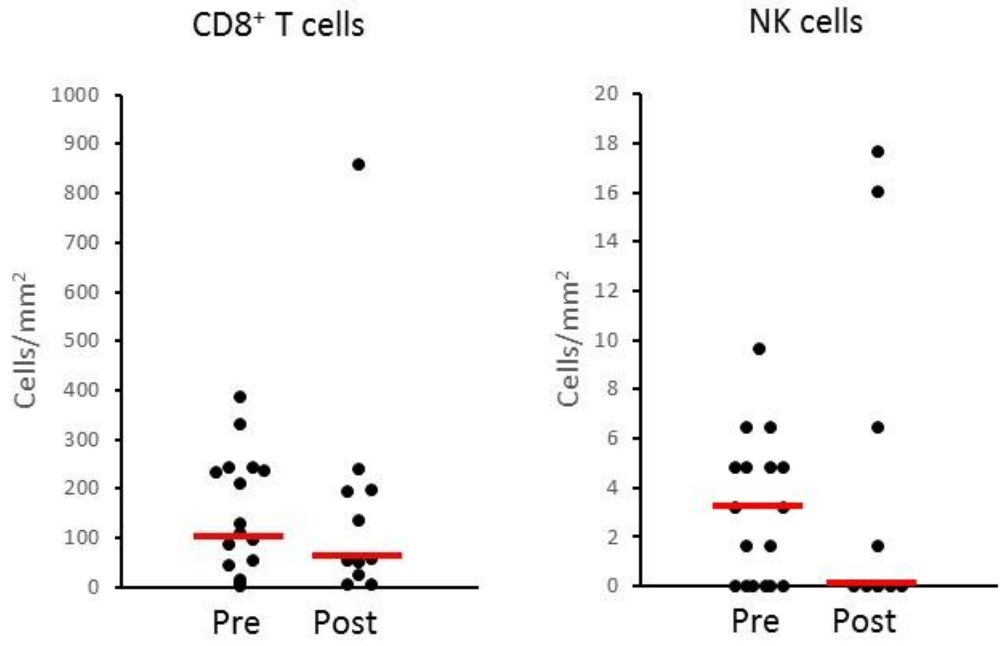

Supplementary Figure 5. Density of $CD8^+$ T cells and $NKp46^+$ cells infiltrating pre-treatment (Pre, n=18) and post-treatment (Post, n=11) metastases from ipilimumab-treated patients. Circles: labeled cell density values of individual samples; horizontal line: median
