# Supplementary material for: HLA Class I Downregulation in Progressing Metastases of Melanoma Patients Treated With Ipilimumab
Source: Pathol Oncol Res. 2022 Apr 22;28:1610297. doi: 10.3389/pore.2022.1610297 (PMC9073691; doi:10.3389/pore.2022.1610297)
Supplement: Supplementary file 3 [file DataSheet1.PDF]

Supplementary Table 1. Patients' clinicopathologic data and immunohistochemistry results

Patient no.	Age	Gender	Stage	ECOG	BRAF	BOR	PFS (mo)	OS (mo)	Sample no.	Location	Time of removal*	HCA2 (%)	HC10 (%)	NAMB-1 (%)	CD8 (cells/mm^2)	NKp46 (cells/mm^2)
Pt1	52	female	III N3c	0	mut	CR	11	67+	Pre1	cut.	-8 months	100,0	100,0	100,0	107,2	6,4
									Pre2	cut.	-6 months	56,6	61,8	74,1	232,0	0,0
									Post1	cut.	+11 months	65,8	72,7	43,7	196,8	16,0
									Post2	sc.	+18 months	83,6	84,2	93,1	857,6	17,6
									Post3	cut.	+18 months	63,5	63,3	51,4	54,4	6,4
Pt2	51	female	IV M1c	0	mut	SD	10	43	Pre1	sc.	-2 months	5,1	4,5	62,2	8,0	3,2
									Post1	sc.	+12 months	11,8	9,8	57,6	4,8	0,0
Pt3	73	male	IV M1a	0	wt	SD	4	42	Pre1	LN	-13 months	87,0	85,0	70,2	241,6	6,4
									Pre2	sc.	-9 months	87,5	43,5	100,0	4,8	4,8
									Pre3	sc.	-9 months	87,5	50,6	97,0	14,4	4,8
									Pre4	sc.	-9 months	93,5	28,7	74,5	1,6	1,6
									Post1	LN	+7 months	74,8	75,5	73,0	193,6	0,0
									Post2	cut./sc.	+7 months	0,0	2,3	2,0	134,4	0,0
Pt4	53	female	IV M1b	0	mut	PD	4	29	Pre1	LN	-23 months	97,5	96,2	87,4	2,9	0,0
									Pre2	sc.	-8 months	100,0	100,0	100,0	84,8	9,6
									Pre3	LN	-2 months	93,6	98,0	100,0	8,0	0,0
									Post1	sc.	+20 months	81,5	67,2	70,4	22,4	0,0
									Post2	sc.	+20 months	77,7	17,5	71,8	49,6	0,0
									Post3	sc.	+20 months	62,7	25,0	61,1	57,6	0,0
Pt5	59	male	IV M1c	1	wt	PD	3	9	Pre1	sc.	-12 months	80,7	68,0	73,0	52,8	3,2
									Post1	cut.	+4 months	0,0	0,0	2,0	4,8	1,6
Pt6	57	female	IV M1c	0	mut	PD	3	8	Pre1	LN	-22 months	79,6	85,7	74,8	128,0	1,6
									Pre2	LN	-22 months	94,4	96,8	92,4	235,2	0,0
									Pre3	LN	-22 months	99,5	100,0	99,5	329,6	4,8
									Pre4	LN	-22 months	100,0	100,0	100,0	243,2	4,8
									Pre5	breast	-13 months	100,0	100,0	100,0	n.e.	n.e.
									Pre6	breast	-13 months	100,0	97,5	72,5	208,0	0,0
									Pre7	breast	-13 months	94,5	84,0	88,5	385,6	0,0
									Post1	LN	+5 months	0,0	0,0	0,0	238,4	6,4

*before or after starting ipilimumab treatment; abbreviations: ECOG: Eastern Cooperative Oncology Group, BOR: best overall response, PFS: progression-free survival, OS: overall survival, Pre: pre-treatment, Post: post-treatment, cut.: cutaneous, sc.: subcutaneous, LN: lymph node
